# β-adrenergic modulation of oddball responses in humans

**DOI:** 10.1186/1744-9081-3-29

**Published:** 2007-06-13

**Authors:** Bryan A Strange, Raymond J Dolan

**Affiliations:** 1 Wellcome Trust Centre for Neuroimaging, Institute of Neurology, 12 Queen Square, London WC1N 3BG, UK; 2 Centre for Clinical Pharmacology and Therapeutics, Division of Medicine, University College London, London, UK

## Abstract

Detection of salient or motivationally significant stimuli is of adaptive importance. The neurophysiological correlates of this detection have been extensively studied in 'oddball' paradigms. Much theoretical data supports the role of noradrenergic systems in generating oddball responses. We combine psychopharmacology and functional neuroimaging to demonstrate modulation of neuronal responses to oddball nouns by the β-adrenergic antagonist propranolol. Critically, responses in regions implicated in oddball detection, namely right ventrolateral prefrontal cortex and temporoparietal junction (TPJ), were abolished by propranolol. Thus, oddball responses depend on modulatory adrenergic inputs, mediated via β-adrenergic receptors.

## Background

Stimuli that violate the prevailing context, oddball stimuli, elicit a P3 event-related potential (ERP). A recent review [1] has highlighted the similarities between conditions evoking the P3 ERP and those evoking phasic responses in the Locus Coeruleus (LC), which provides the major ascending noradrenergic (NE) projection. Early studies combining animal intracranial recordings, lesion studies and human scalp-recorded ERPs provided evidence for the involvement of the LC-NE system in P3 generation [1,2]. The human electrophysiological response to oddball stimuli (P3) is dependent on the local and global probability of their occurrence [3,4]. In addition, P3s are elicited by motivationally significant stimuli, such as emotional stimuli [5]. In parallel, data from animal studies demonstrate increased phasic activity in LC neurones to novel, infrequent or threatening stimuli [6,7].

Thus, in the context of fMRI scanning, we presented subjects with two types of infrequent oddballs, perceptual and emotional, both known to elicit a P3 [1,3,5] and to engage LC in animals [1,6,7]. To examine the role of the β-adrenergic system in oddball responses, 24 right-handed, native English speaking subjects were scanned in a double-blind placebo-controlled design with 12 subjects receiving a 40 mg dose of propranolol and 12 placebo. We scanned subjects while viewing nouns presented visually at a rate of one every 3 s. Subjects were presented with 38 lists of 14 nouns and were required to indicate with a push-button whether or not the first letter in the noun had an enclosed space (shallow encoding). For each list, 12 nouns were of the same semantic category, were emotionally neutral and presented in the same font, *i.e*. neutral control (C) nouns. A perceptual (P) oddball was presented in a novel font but was emotionally neutral and of the same semantic category as the neutral nouns. An emotional (E) oddball was aversive in content but of the same category and perceptually identical to neutral nouns. Thus, both oddball types had a frequency of 1 in 14 (global probability of occurrence 0.07). The first 5 nouns in each list were always neutral nouns, engendering low local probability of oddball occurrence.

## Materials and methods

### Subjects

The 24 subjects comprised 12 males (age range 20–39 yrs; mean 29.2) and 12 females (20–29 yrs; 24.7) with drug allocation balanced for gender *i.e*. 6 males and 6 females received propranolol. All subjects gave informed consent and were free of neurological or psychiatric history. The study had full ethics approval.

### Task

Subjects were administered propranolol or placebo at 0730. In view of the kinetics of propranolol's peak plasma concentration (1–2 h), the oddball task started 90 min after drug administration. Blood pressure (BP) was measured at the time of drug/placebo administration and blood samples and BP were taken immediately prior to the encoding scanning session [8].

Nouns were presented every 3 s (stimulus duration 1 s) in lower case Times font (48 point; 4–10 degrees of horizontal visual angle) except for perceptual oddballs, which appeared in 19 different fonts. Each list of 14 nouns was separated by presentation of the words 'New List'. Instructions for shallow encoding were provided visually at the start of each session. The 38 lists were normed for semantic relatedness and emotional valence by a separate group of 12 subjects [5 male (24–37 yrs; 28.2); 7 female (23–37 yrs; 27.9)]. Control nouns, like oddballs, could not occur within the first 5 nouns of each list and could not immediately follow an oddball or another chosen control noun.

In discussing our oddball-induced activations we refer to the P3 ERP. The P3 complex has been divided on the basis of scalp topography and task correlates into the fronto-central P3a, evoked by novel distractor stimuli and a component of the characteristic response to orienting stimuli, and a later parietal P3b, evoked by infrequent target stimuli. Our experiment was not designed to dissociate these two components.

### Data Acquisition

A 2T Siemens VISION system (Siemens, Erlangen) was used to acquire both T1-weighted anatomical images and gradient-echo echo-planar T2*-weighted MRI image volumes with blood oxygenation level dependent (BOLD) contrast. Volumes were acquired continuously every 2506 ms, with a total of 690 volumes acquired per subject. Each volume comprised 33 3.3 mm axial slices, with an in-plane resolution of 3 × 3 mm, positioned to cover the cerebrum. Imaging time series were then realigned to correct for interscan movement, slice-time corrected, normalised into a standard anatomical space, and smoothed with a Gaussian kernel of 6 mm full width half-maximum [9].

### Data Analysis

Imaging data were analysed using Statistical Parametric Mapping (SPM99) employing an event-related model with a two stage random effects procedure. The effects of interest were the events corresponding to the E and P nouns and the two randomly selected control nouns. Trial-specific responses were modelled by convolving a delta function that indicated each event onset with a synthetic, canonical haemodynamic response function (HRF) to create regressors of interest. All other nouns, the presentation of the "New List" marker, as well as oddballs or selected control nouns for which encoding responses were incorrect or absent, were modelled separately. Movement parameters and low frequency drifts in signal (cut-off 120 secs) were modelled as nuisance covariates. For the drug group, data from one subject was not included because of poor image quality.

From the 1^st ^level analysis described above, session-specific parameter estimates of the magnitude of the haemodynamic response for each stimulus type were calculated for each voxel in the brain [10]. A contrast of parameter estimates modelling each comparison of interest (*e.g*. emotional oddballs vs control nouns) was calculated in a voxel-wise manner to produce, for each subject, one contrast image for that particular effect. Two contrast images for each subject were carried forward to the random effects analysis: perceptual and emotional oddballs versus their respective controls were entered into an ANOVA (corrected for non-sphericity) across the 12 placebo and propranolol subjects. We employed a conjunction analysis to test for activation common to both perceptual and emotional oddballs versus their respective controls under placebo as well as for the placebo vs drug comparison. A conjunction analysis tests for a significant main effect in the absence of any interactions among the simple effects. [11]. The whole-brain statistical parametric maps that ensued from the conjunction analyses were thresholded at p < 0.001 uncorrected and examined for evidence of activation. We report P values of the ensuing maxima that survive threshold at p < 0.001 (uncorrected), except for bilateral supramarginal gyrus, which we report descriptively.

## Results

**Figure 1:**
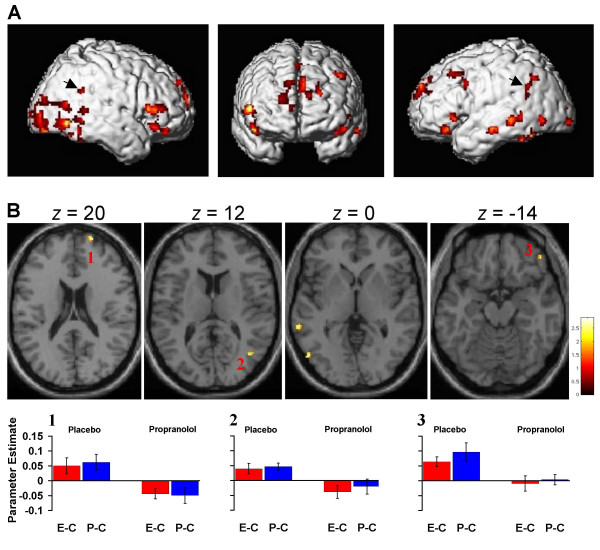
Oddball responses in humans are modulated by the β-adrenergic antagonist propranolol. (**a**) Oddball responses in the placebo group common to both emotional (E) and perceptual (P) oddballs. The statistical parametric map (SPM) is rendered onto a canonical brain. The threshold for illustration, here and in (b), is p < 0.005, uncorrected, with an extent threshold here of 25 voxels. Arrows point to responses in bilateral supramarginal gyrus. (**b**) Oddball-evoked responses that are significantly greater in the placebo vs propranolol group. The SPM (masked inclusively by regions showing oddball responses in the placebo group) is overlaid on axial slices with the *z *coordinate (distance in mm from the anterior commissure) indicated above. Response estimates are plotted relative to control (C) nouns for the response in *1 *right anterior prefrontal cortex, *2 *right temporo-parietal junction, and *3 *right inferior frontal gyrus (co-ordinates given in table 1). Error bars represent ± 1 SE of the mean.

Oddball responses are expressed relative to 2 randomly-selected neutral nouns (one for each oddball type) presented at encoding. Different control nouns were chosen for each oddball to provide independent baselines and permit analyses of evoked neuronal responses common to both oddball types. We first tested for regions commonly activated by perceptual and emotional oddballs relative to their respective controls in the placebo group. As shown in Fig. 1a, a random effects analysis of this comparison yielded activation in a network including prefrontal, parietal, temporo-occipital and fusiform cortex. Emotional oddballs engaged bilateral anterior temporal and left inferior frontal cortex to a greater extent than perceptual oddballs (as well as left amygdala at an uncorrected threshold of p < 0.005). By contrast, the P vs E noun comparison demonstrated bilateral occipito-parietal activation extending into inferior temporal cortex.

We next compared oddball-evoked responses between groups. Fig. 1b (also see table 1) shows responses significantly attenuated by propranolol relative to the placebo group. Oddball responses in bilateral occipitotemporal cortex, right TPJ, right ventrolateral and right anterior prefrontal cortex were abolished by β-adrenergic blockade. Plots of the response estimates clearly demonstrate that activations in regions previously shown to respond to oddballs are engaged by both oddball types and that these responses are selectively abolished by propranolol. Fig. 1a demonstrates oddball-evoked responses in a more superior locus of TPJ bilaterally, in supramarginal gyrus (arrowed). This locus, strongly implicated in oddball detection [14], showed significantly reduced activation bilaterally by propranolol, albeit at a lower threshold.

## Discussion

Previous investigation of NE modulation of oddball responses in humans has been limited in two respects; 1) pharmacological studies have been limited to scalp-recorded ERPs, which provide limited anatomical specificity, 2) studies have used α2-adrenergic receptor agonists such as clonidine, which, in addition to causing sedation, act on presynaptic autoreceptors to inhibit noradrenaline release. Thus the postsynaptic target receptors mediating NE effects are unknown, with further uncertainty arising from the fact that clonidine also inhibits release of 5-hydoxytryptamine (5-HT) [12]. Despite these potential limitations, our findings accord with a previous study demonstrating a reduction of TPJ activity in response to an alerting cue by the α2-agonist clonidine [13].

Our data are supported by prior studies of oddball response generators as well as the distribution of NE cortical innervation. It is thought that detection of behaviourally relevant stimuli, particularly when salient, engages a largely right lateralized temporoparietal and ventral prefrontal (including inferior and middle frontal gyri and operculum) cortical network [14]. Scalp-recorded ERPs in brain damaged patients [15], intracranial recordings from patients with implanted electrodes [16] as well as neuroimaging studies [14] have demonstrated oddball-evoked responses in TPJ and prefrontal cortex, particularly in the region of inferior frontal sulcus. NE projections are most dense to inferior parietal cortex, somatosensory cortex, frontal pole and TPJ (see [1] and [10] for review). Those regions showing oddball responses in the placebo group, but which were not modulated by drug, namely fusiform cortex, receive relatively weak NE projections.

## Conclusion

The areas within which we demonstrate modulation of oddball-evoked responses overlap strongly with regions implicated in oddball detection and with projections of the LC-NE system. We suggest that our data provide direct empirical support for a neurophysiological model that oddball responses are mediated via the NE system [1], via β-adrenergic receptors.

## Competing interests

The author(s) declare that they have no competing interests.

## Authors' contributions

BAS and RJD designed the study and wrote the manuscript. BAS acquired and analysed the data. All authors read and approved the final manuscript.

**Table 1:** Placebo vs Propranolol *p < 0.001 uncorrected;^† ^p < 0.05 uncorrected

**Brain area**	**Coordinates (*x*, *y*, *z*)**	***Z *score**
Right anterior prefrontal cortex	22, 64, 20	4.27*
Left occipito-temporal cortex	-52, -70, 0	4.00*
Right occipito-temporal cortex	50, -62, -4	3.62*
Left posterior inferior temporal sulcus	-62, -38, 0	3.95*
Right temporo-parietal junction	42, -68, 12	3.54*
Right inferior frontal gyrus	48, 44, -14	3.47*
Right supramarginal gyrus	58, -46, 28	2.21^†^
Left supramarginal gyrus	-48, -56, 34	2.43^†^

## References

[B1] Nieuwenhuis S, Aston-Jones G, Cohen JD (2005). Decision making, the P3, and the locus coeruleus-norepinephrine system. Psychol Bull.

[B2] Pineda JA, Westerfield M (1993). Monkey P3 in an "oddball" paradigm: pharmacological support for multiple neural sources. Brain Res Bull.

[B3] Duncan-Johnson CC, Donchin E (1977). On quantifying surprise: the variation of event-related potentials with subjective probability. Psychophysiol.

[B4] Squires KC, Wickens C, Squires NK, Donchin E (1976). The effect of stimulus sequence on the waveform of the cortical event-related potential. Science.

[B5] Keil A, Bradley MM, Hauk O, Rockstroh B, Elbert T, Lang PJ (2002). Large-scale neural correlates of affective picture processing. Psychophysiol.

[B6] Foote SL, Aston-Jones G, Bloom FE (1980). Impulse activity of locus coeruleus neurons in awake rats and monkeys is a function of sensory stimulation and arousal. Proc Natl Acad Sci USA.

[B7] Rasmussen K, Morilak DA, Jacobs BL (1986). Single unit activity of locus coeruleus neurons in the freely moving cat. I. During naturalistic behaviors and in response to simple and complex stimuli. Brain Res.

[B8] Strange BA, Dolan RJ (2004). Beta-adrenergic modulation of emotional memory-evoked human amygdala and hippocampal responses. Proc Natl Acad Sci USA.

[B9] Friston KJ, Ashburner J, Frith CD, Poline J-B, Heather JD, Frackowiak RSJ (1995). Spatial registration and normalisation of images. Hum Brain Mapp.

[B10] Friston KJ, Homes AP, Worsely KJ, Poline J-B, Frith CD, Frackowiak RSJ (1995). Statistical parametric maps in functional imaging: a general linear approach. Hum Brain Mapp.

[B11] Price CJ, Friston KJ (1997). Cognitive conjunction: A new approach to brain activation experiments. NeuroImage.

[B12] Scheibner J, Trendelenburg AU, Hein L, Starke K (2001). Alpha2-adrenoceptors modulating neuronal serotonin release: a study in alpha2-adrenoceptor subtype-deficient mice. Br J Pharmacol.

[B13] Coull JT, Nobre AC, Frith CD (2001). The noradrenergic alpha2 agonist clonidine modulates behavioural and neuroanatomical correlates of human attentional orienting and alerting. Cereb Cortex.

[B14] Corbetta M, Shulman GL (2002). Control of goal-directed and stimulus-driven attention in the brain. Nat Rev Neurosci.

[B15] Yamaguchi S, Knight RT (1991). Anterior and posterior association cortex contributions to the somatosensory P300. J Neurosci.

[B16] Baudena P, Halgren E, Heit G, Clarke JM (1995). Intracerebral potentials to rare target and distractor auditory and visual stimuli. III. Frontal cortex. Electroenceph Clin Neurophysiol.

